# Author Correction: A fluorogenic, peptide-based probe for the detection of Cathepsin D in macrophages

**DOI:** 10.1038/s42004-025-01872-w

**Published:** 2026-01-10

**Authors:** Maria Rodriguez-Rios, Brian J. McHugh, Zhengqi Liang, Alicia Megia-Fernandez, Annamaria Lilienkampf, David Dockrell, Mark Bradley

**Affiliations:** 1 School of Chemistry, University of Edinburgh, David Brewster Road, EH9 3FJ Edinburgh, UK; 2 University of Edinburgh Centre for Inflammation Research, Queen’s Medical Research Institute, 47 Little France Crescent, Edinburgh BioQuarter, Edinburgh, EH16 4TJ UK; 3 Organic Chemistry Department, Faculty of Sciences, University of Granada, Avda. Fuente Nueva S/N, Granada, 18071 Spain; 4 Precision Healthcare University Research Institute, Queen Mary University of London, Empire House, 67-75 New Road, London, E1 1HH UK

**Keywords:** Chemical tools, Peptides, Proteases, Fluorescent probes, Imaging studies

Correction to: *Communications Chemistry* 10.1038/s42004-023-01035-9, published online 2 November 2023

In the version of this article initially published, there were errors in the presentation of structures shown in Fig. 1, where the CatD-P3 probe structure was missing a carbon atom in the PEG tag, where the propargyl moiety in alkyne-PEG(5 K)-OMe used for the click chemistry was previously depicted as an ethynyl moiety. In Fig. 2 and Supplementary Note 4, the PEG reagent used to synthesize CatD-P3 was similarly depicted incorrectly. In addition, in Fig. 2, the charges of structure 2 were erroneously misplaced within the BODIPY fluorophore. The errors were visual and do not affect the results or conclusions in the article. Figures 1 and 2 and Supplementary Note 4 have now been updated in the HTML and PDF versions of the article.

