# Supplementary material for: A fluorogenic, peptide-based probe for the detection of Cathepsin D in macrophages
Source: Commun Chem. 2023 Nov 2;6:237. doi: 10.1038/s42004-023-01035-9 (PMC10622513; doi:10.1038/s42004-023-01035-9)
Supplement: Supplementary file 2 — Supplementary Information [file 42004_2023_1035_MOESM2_ESM.pdf]

Supplementary Information

A Fluorogenic, Peptide-Based Probe for the Detection of Cathepsin D in Macrophages

Maria Rodriguez-Rios,[a] Brian J. McHugh,[b] Zhengqi Liang,[a] Alicia Megia-Fernandez,[a,c] Annamaria Lilienkampf,[a] David Dockrell[b] and Mark Bradley[d,*]

[a]*EaStCHEM School of Chemistry, University of Edinburgh, David Brewster Road, EH9 3FJ Edinburgh, U.K.;*

[b]*University of Edinburgh Centre for Inflammation Research, Queen's Medical Research Institute, 47 Little France Crescent, Edinburgh BioQuarter, Edinburgh EH16 4TJ, U.K.;*

[c]*Organic Chemistry Department, Faculty of Sciences, University of Granada, Avda. Fuente Nueva S/N, 18071 Spain.*

[d]*Precision Healthcare University Research Institute, Queen Mary University of London,* Empire House, 67-75 New Road, London E1 1HH

* m.bradley@qmul.ac.uk

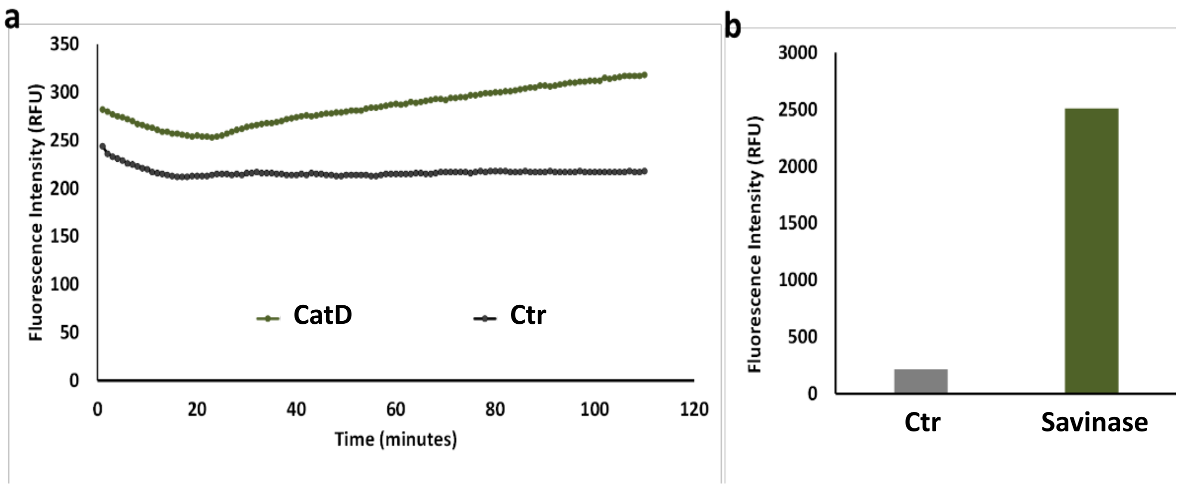

Supplementary Figure 1. A) **CatD-P1** (20 µM in 10% DMSO/50 mM NaOAc, pH 4 did not show a significant increase in fluorescence when incubated with CatD (up to 300 nM or 4U/mL); b) **CatD-P1** (20 µM) exposure to Savinase (a broad-spectrum protease, 0.4U/mL) resulted in probe activation and notable increase in fluorescence (plate reader fluorescence gain = 35). Ctr = **CatD-P1** probe only. Note: the Savinase used is stored in glycerol, which may have aided probe solubility in the control experiment.

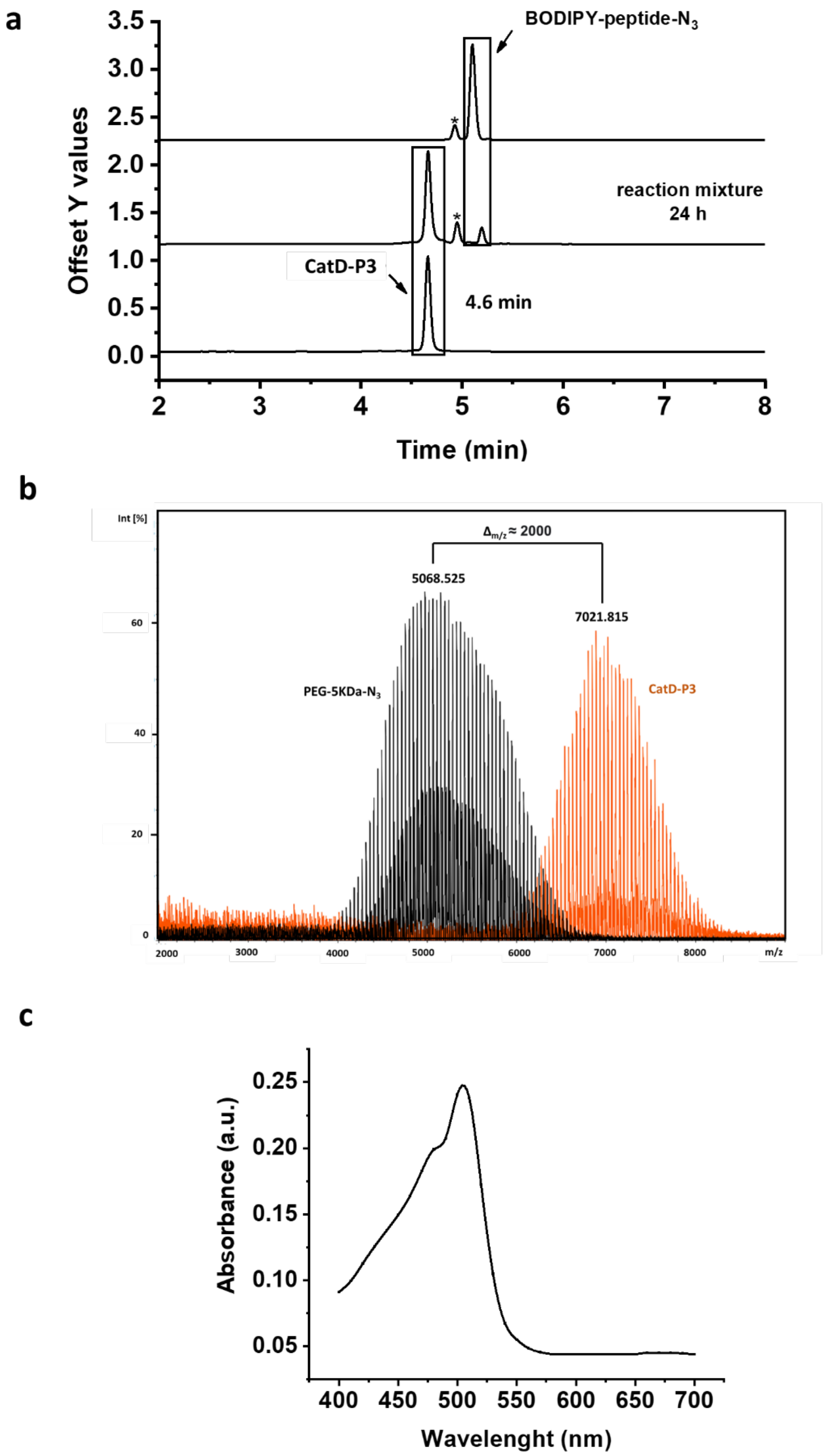

Supplementary Figure 2. Reaction monitoring and characterisation of probe **CatD-P3.** a) The CuAAC reaction was monitored by RP-HPLC (495 nm) over 6 min using a gradient of ACN/H_2O 0.1% FA. * Indicates the peak for the unconjugated BODIPY-COOH fluorophore from the previous reaction; b) MALDI-TOF MS analysis of the 5 KDa PEG-alkyne (black spectrum) and the purified **CatD-P3** probe (red spectrum) (average mass found (m/z) Mn: 7096.471); c) Absorption spectrum of **CatD-P3** (in sterile water).

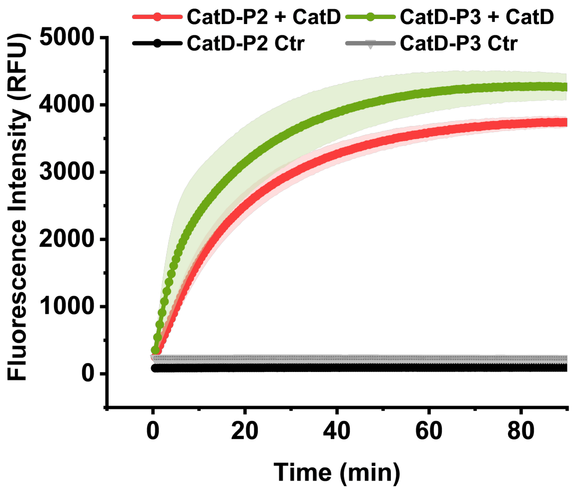

Supplementary Figure 3. Comparison of **CatD-P2** and **CatD-P3** (20 µM) activation in the presence or absence of CatD (50 nM) (enzyme reaction buffer 50 mM NaOAc pH 4). **CatD-P2** and **CatD-P3** stocks were prepared (60 µM in 10 % DMSO in water or 100% water, respectively) and diluted with enzyme reaction buffer to a final concentration of 20 µM. Probes were exposed to CatD (50 nM) and the increase in fluorescence (λ_{ex} 485/20 nm λ_{em} 520/20 nm) was monitored over 1.5 h. Both probes showed comparable activation profiles. The **CatD-P2** assay required the addition of 10 % DMSO for probe solubility. Measurements were taken in triplicate from distinct samples and mean values and standard deviation error bars were calculated and plotted using OriginLab software.

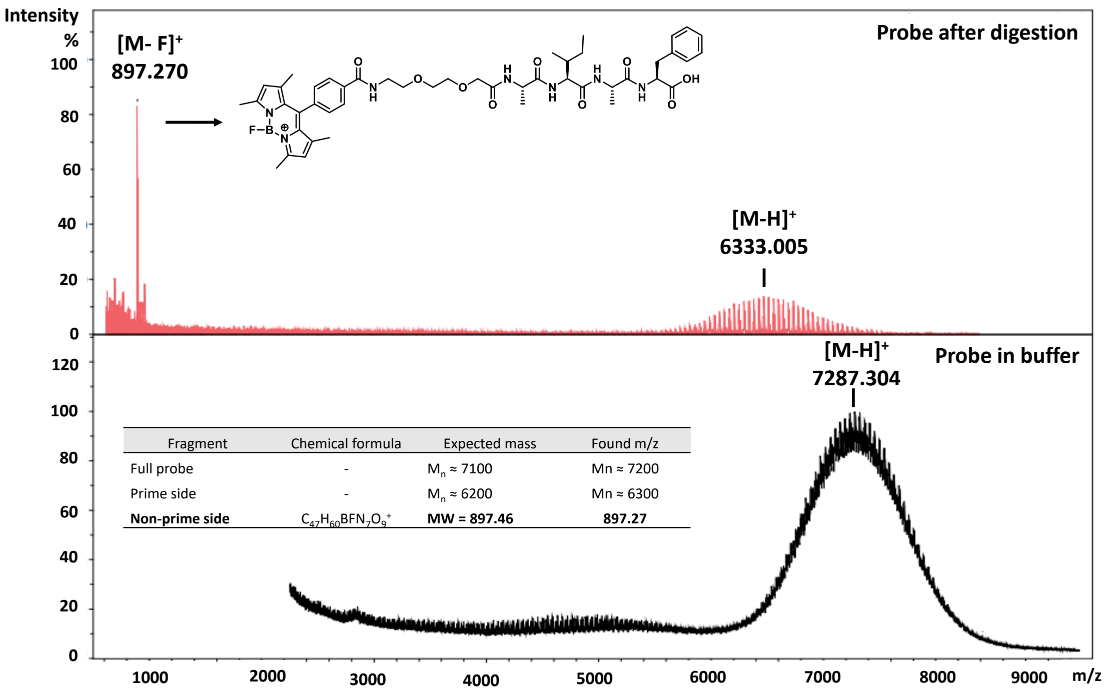

Fragment	Chemical formula	Expected mass	Found m/z
Full probe	-	$M_n \approx 7100$	Mn ≈ 7200
Prime side	-	$M_n \approx 6200$	Mn ≈ 6300
**Non-prime side**	$C_{47}H_{60}BFN_7O_9^+$	**MW = 897.46**	**897.27**

Supplementary Figure 4 MALDI-TOF MS analysis following exposure of **CatD-P3** to Cathepsin D. The N-terminal BODIPY containing fragment confirmed cleavage happened between the two phenylalanine residues. The mass ion showed a loss of fluoride from the BODIPY fluorophore$[M-F]^+$ Expected: 897.46 found: 897.27. Specific hydrolytic cleavage between Phe↑Phe was confirmed following probe (10 µM) incubation with Cat D (50 nM) for 90 min at 37 ℃ and analysis of the reaction mixture by MALDI-TOF MS with the appearance of mass peaks of the expected sizes. The BODIPY fragment ion showed a loss of -19, consistent with the loss of a fluorine ion $[M-F]^+$ from the boronate salt[1] (this ion was not observed when the compound was analysed by LC-MS,

suggesting this is a result of the ionisation method, which is consistent with previously reported analysis of BODIPY dyes by mass spectrometry[1]).

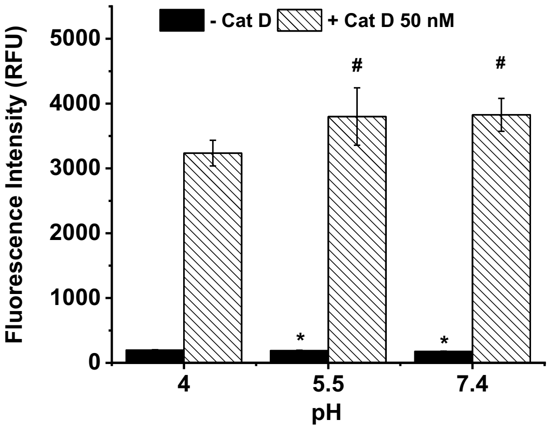

Supplementary Figure 5. The effect of pH on the fluorescence signal. **CatD-P3** (60 µM) was incubated 1.5 h in the reaction buffer (50 mM NaOAc pH 4.0) in a final volume of 200 µL with or without CatD (50 nM, Athens Biotechnology). The solution was then diluted in the corresponding buffer (pH buffers (50 mM NaOAc pH 4.0 or 5.5, PBS pH 7.4) to give final concentrations in the well of 12 µM (50 µL volume) and fluorescent intensities were measured using a fluorescence microplate reader (Biotek Synergy HT multi-mode reader) ($\lambda_{ex/em}$ 485/20, emission 520/20) at 37 °C. Two tailed paired Student's test statistical analysis showed no significant differences (p-values > 0.05) in data comparisons where asterisk (*) refers to values compared to uncleaved probe at pH 4 and dash (#) refers to values compared to fully cleaved probe at pH 4. Measurements were taken in triplicate from distinct samples and mean values and standard deviation error bars were calculated and plotted using OriginLab software.

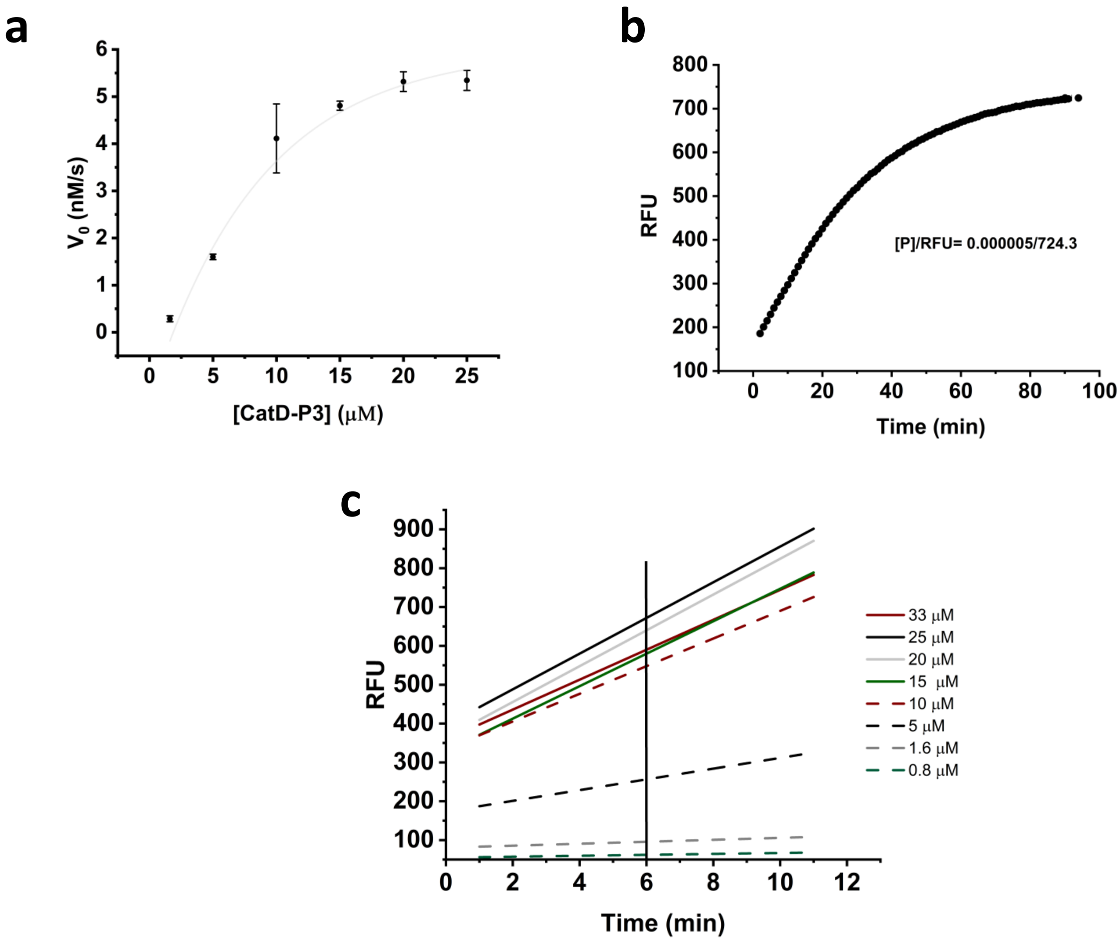

Supplementary Figure 6. Kinetic parameter determination for **CatD-P3** a) Michaelis-Menten curve built from exposing increasing concentrations of the probe to CatD (10 nM). b) Normalisation factor ([P]/RFU_{max}) was obtained from 5 µM activation of the probe (where RFU_{max} was 1484). c) Initial velocity plots showing fluorescence increases linearly over the first 10 min of the reaction, the vertical line shows the cut-off time for calculations (10 min). Values of fluorescence were obtained in triplicates on a Biotek Synergy HT multi-mode reader, with 1 min intervals over 5 min and λ_{ex} = 485/20, λ_{em} = 528/20 (with a fluorescence gain of 50). Measurements were taken in triplicate from distinct samples and mean values and standard deviation error bars were calculated and plotted using OriginLab software.

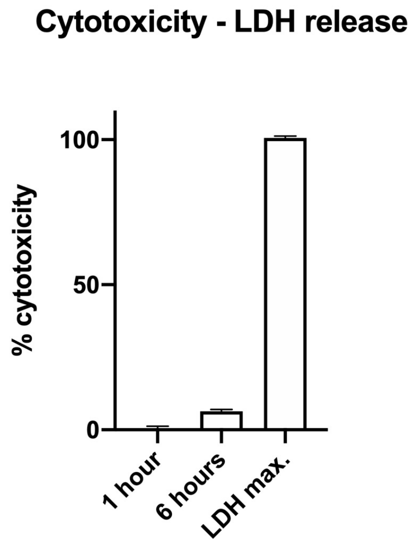

Supplementary Figure 7. Cytotoxicity of probe **CatD-P3** on monocyte derived macrophages (MDM), assessed by LDH release. After incubation with 10 µM CatD-P3, supernatant from MDMs was monitored at 1 and 6 h and compared to maximum LDH release from lysed cells. LDH release is shown as a percentage of maximum LDH release detected by this assay, +/- SEM (n = 3 assays). Statistical analysis was by paired t-test, performed with GraphPad Prism software.

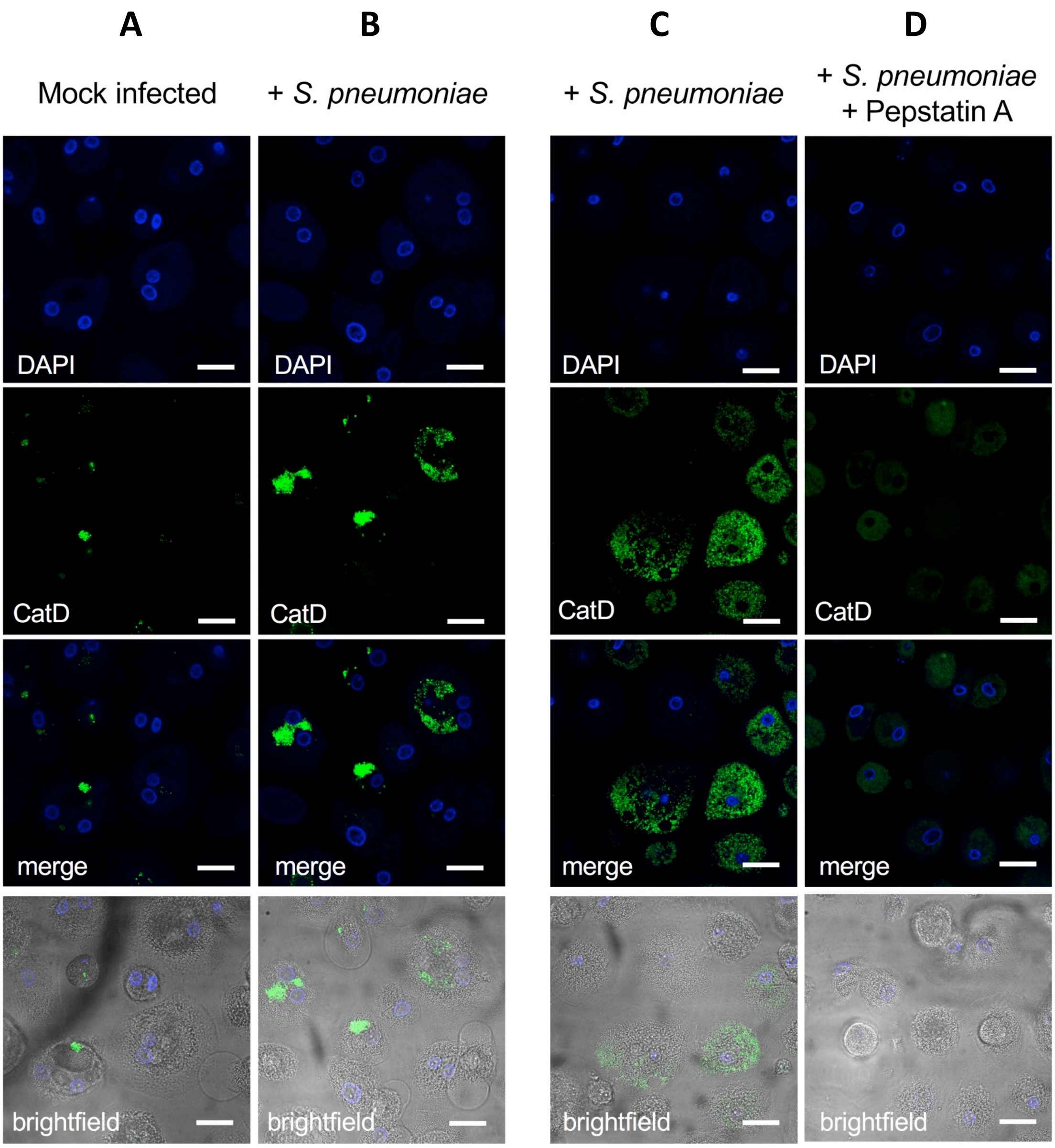

Supplementary Figure 8. Confocal fluorescence microscopy (λ_{ex} 488, λ_{em} 512 nm) images showing the detection of CatD activity in monocyte-derived macrophages challenged with bacteria. **(A)** Mock infected macrophages (no bacteria) showing negligible levels of fluorescence (in the presence of **CatD-P3** (10 µM). (**B**) showing CatD release from lysosomes into the cell cytoplasm induced by challenging macrophages with *S. pneumoniae* in the presence of **CatD-P3** (10 µM). (**C**) is a replicate of the conditions in (**B**) and **(D)** shows how the addition of the CatD inhibitor Pepstatin A (20 µM) prevented activation of CatD-P3 in infected macrophages. In these experiments, macrophages were incubated for 6 h in the presence or absence (Mock) of *S. pneumoniae* (at a Multiplicity of Infection of 10:1), while in the presence of **CatD-P3** (10 µM) Pepstatin A (20 µM) was added after 4 h. At 10 h the cells were washed, fixed in 2% paraformaldehyde and cell nuclei were counter-stained with DAPI (λ_{ex} 405, λ_{em} 461 nm). Scale bar = 10 µm. In the images, cells appear phenotypically normal, and the DAPI stained nuclei show no signs of apoptosis.

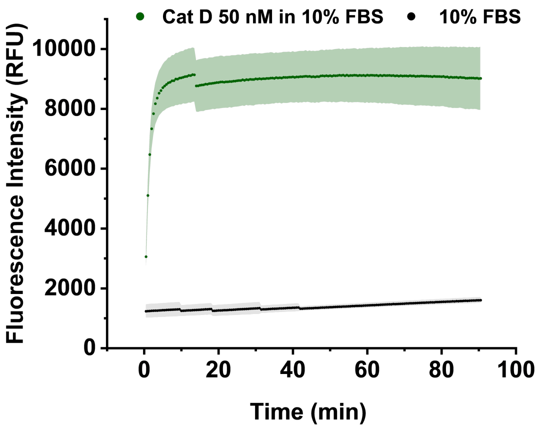

Supplementary Figure 9. Time dependent activation of **CatD-P3** in reaction buffer and in pH 4.0 adjusted 10% FBS over 1 h. Measurements were taken in triplicate from distinct samples and mean values and standard deviation error bars were calculated and plotted using OriginLab software.

Compound Characterisation

Supplementary Note 1. CatD-P1 characterisation. **LC-MS (ESI)**: $[M+3H]^{3+}$ 563.3; **FT-HRMS:** Calculated m/z for $C_{86}H_{114}BF_2N_{19}O_{14}^+$ $[M+H]^+$ 1685.88073; Found: 1685.87628. **HPLC** (495 nm, method A): $\mathbf{t_R}$ of 4.9 min; $\boldsymbol{\lambda_{abs}}$ 500 nm (max) $\boldsymbol{\lambda_{em}}$ 520 nm (max).

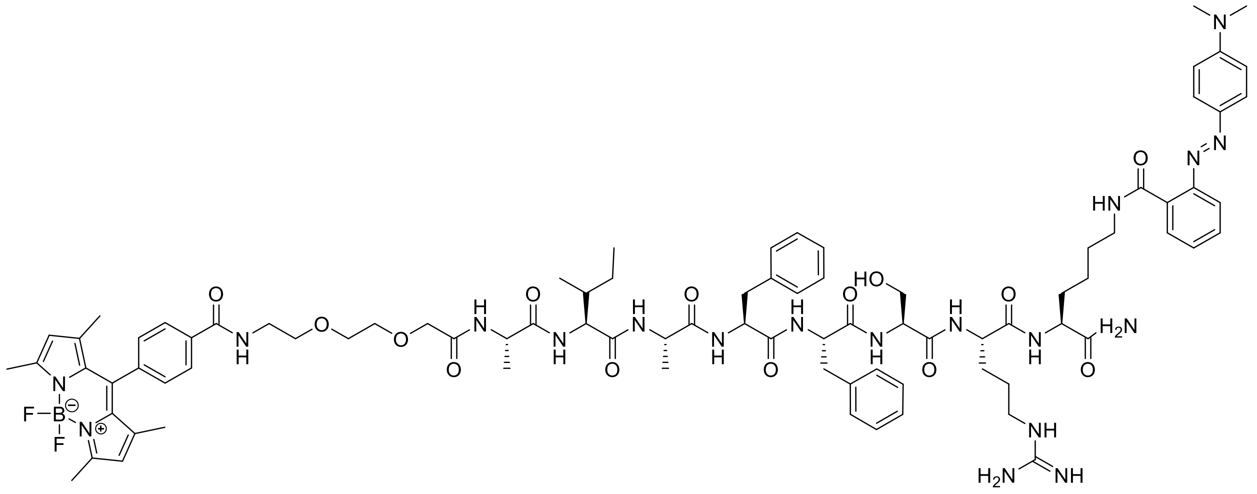

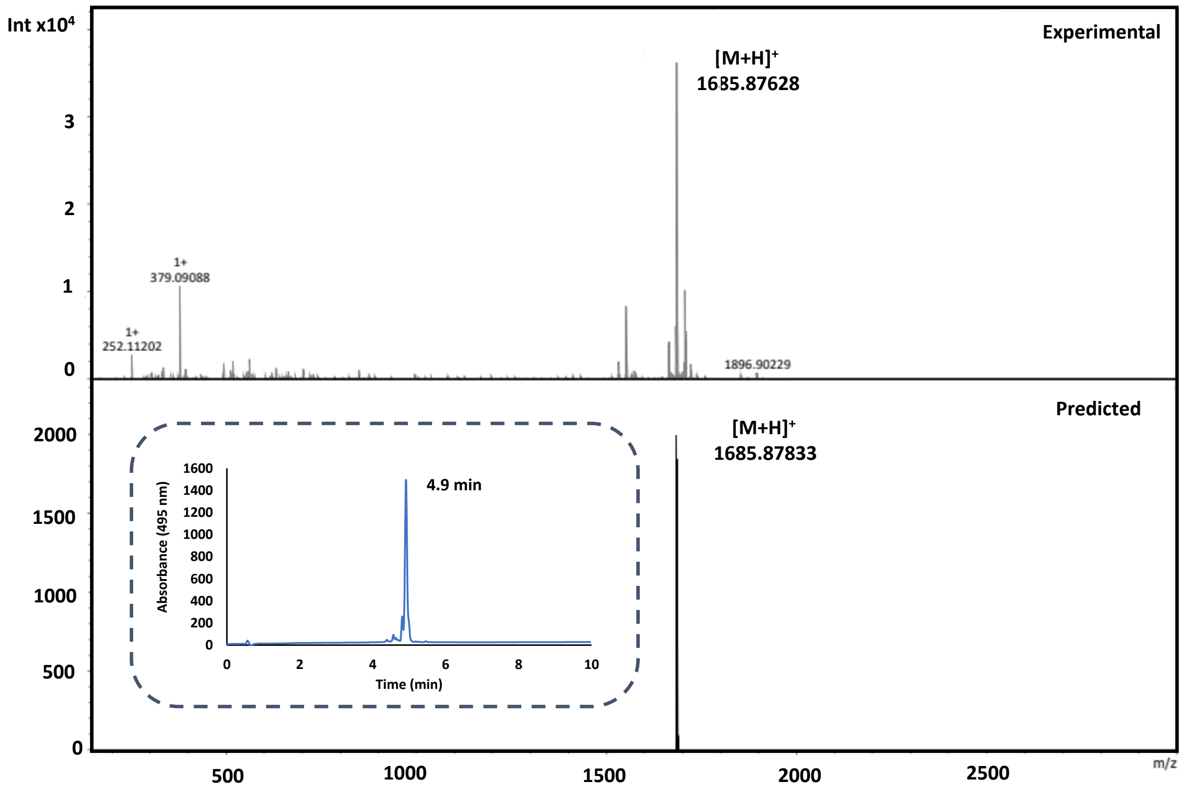

Supplementary Figure 10. CatD-P1 characterisation. FT-HRMS and RP-HPLC analysis (495 nm) for **CatD-P1**.

Supplementary Note 2. CatD-P2 characterisation. LC-MS (ESI) $[M+2H]^{2+}$ 1060.2; **FT-HRMS:** Calculated m/z for $C_{104}H_{146}BF_2N_{22}O_{23}$ $[M+H+K]^{+2}$ 1079.5260; Found: 1079.5309; **HPLC** (495 nm, method B): $\mathbf{t_R}$ of 4.8 min, **HPLC** (495 nm, method B): $\mathbf{t_R}$ of 6.7 min, purity 98 %; λ_{abs} 500 nm (max) λ_{em} 520 nm (max).

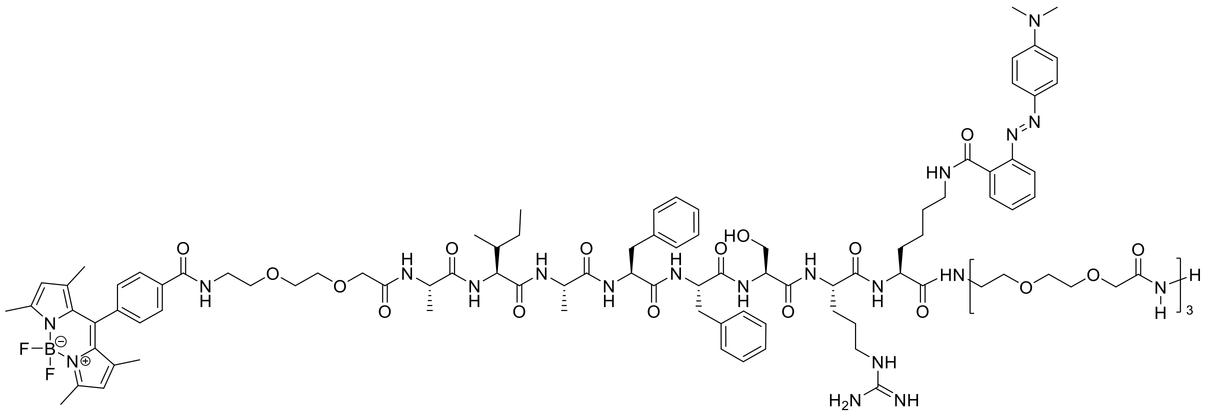

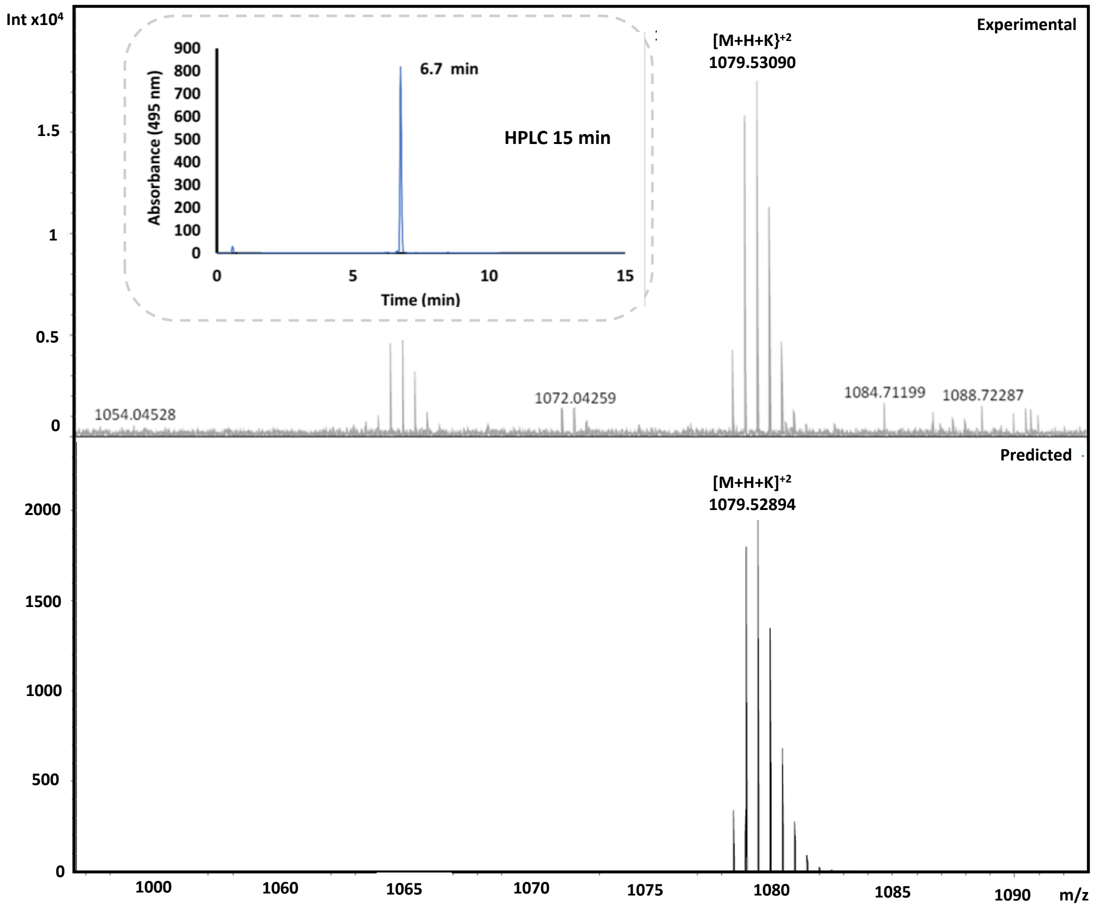

Supplementary Figure 11. CatD-P2 characterisation. FT-HRMS and RP-HPLC analysis (495 nm) for CatD-P2. The HPLC shows a trace of DMSO peak at 0.5 min.

Supplementary Note 3. Azide-BODIPY-peptide (5) characterisation. LC-MS (ESI) $[M+2H]^{2+}$: 655.4 **FT-HRMS:** Calculated m/z for $C_{98}H_{134}BF_2N_{24}O_{18}$ $[M+H]^{+}$ 1985.0401, found: 1985.0386. **HPLC-UV** (495 nm, method A): $\mathbf{t_R}$ of 5.2 min, purity 86 % $\boldsymbol{\lambda_{abs}}$ 500 nm (max) $\boldsymbol{\lambda_{em}}$ 520 nm (max)

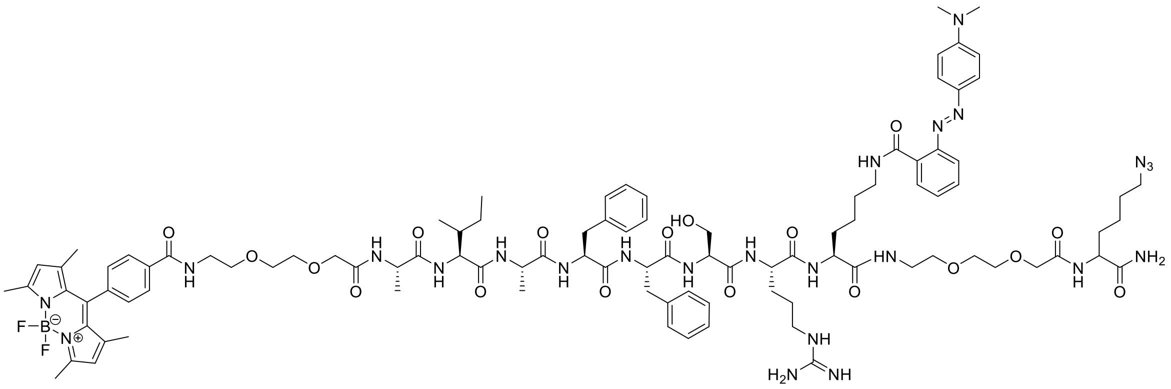

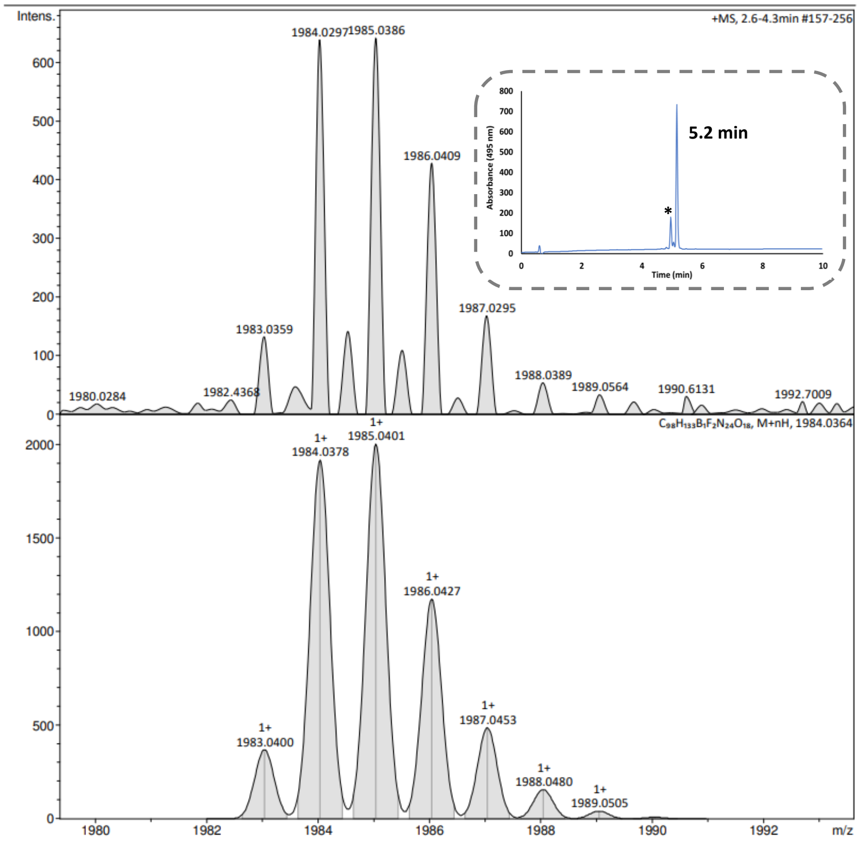

Supplementary Figure 12. Azide peptide 5 characterisation. FT-HRMS results (up: experimental, down: theoretical) and RP-HPLC analysis (495 nm). * indicates residual BODIPY-COOH (14%).

Supplementary Note 4 CatD-P3 characterisation. HPLC-UV (495 nm, method A): **t_R** of 4.6 min, purity >99 %, **λ_{abs}** 500 nm (max), **λ_{em}** 520 nm (max). **MALDI-TOF MS:** m/z found 6000-8000, the spectrum shows a broad mass distribution due to the polydisperse PEG unit.

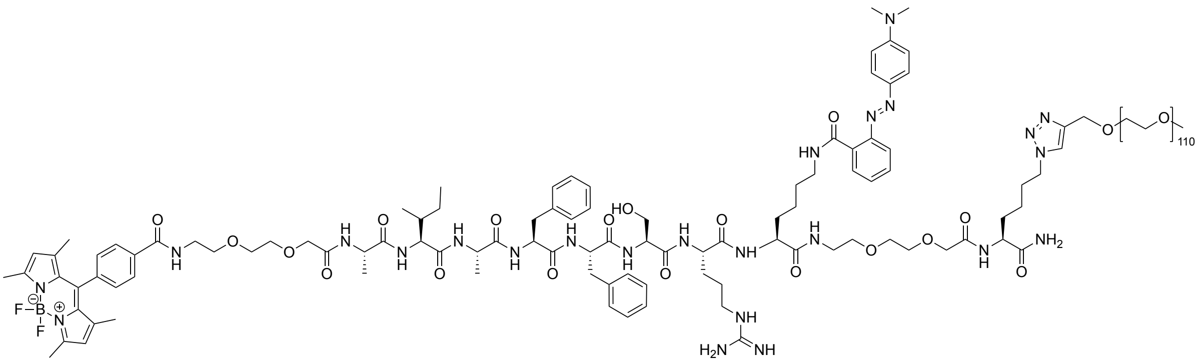

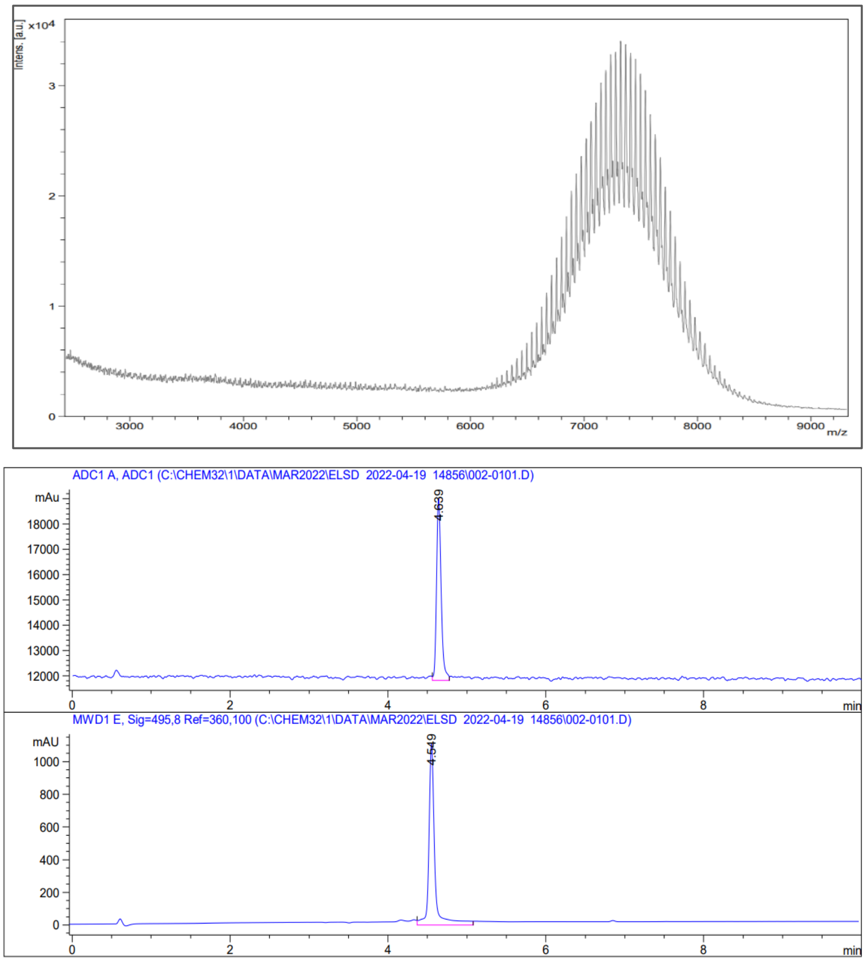

Supplementary Figure 13. **CatD-P3 MALDI-TOF MS** spectrum for $[M+H]^+$, m/z found 6000-8000 and **HPLC traces (Method A at 495 nm or ELSD)** : t_R 4.6 min.

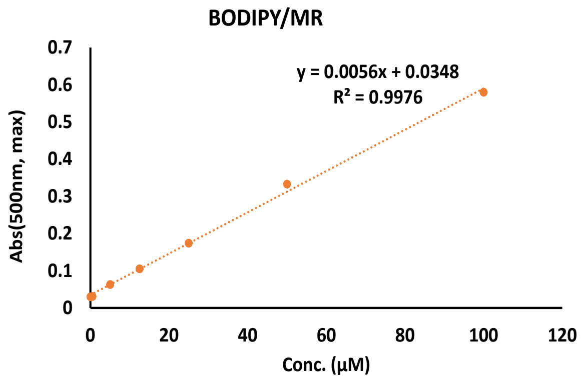

Supplementary Figure 14. A calibration line for the calculation of the concentration of probes **CatD-P1**, **CatD-P2** and **CatD-P3** used to determine the absolute probe concentration. Absorbance values were plotted against concentration for 1:1 solution of BODIPY/MR. Solutions (50 µL) of increasing concentration were added to a 96-well plate and 500 nm absorbance values were obtained using a plate reader (Biotek HT Synergy). Measurements were taken in triplicate from distinct samples and mean values and standard deviation error bars were calculated and plotted using Excel.

Supplementary references

1. ÇetİndereS, YeŞİlot S, KiliÇ A. Pyrene-BODIPY-substituted novel water-soluble cyclotriphosphazenes: synthesis, characterization, and photophysical properties. *Turk J Chem* 2020, **44**(1)**:** 1-14.
