# Supplementary material for: A fluorogenic, peptide-based probe for the detection of Cathepsin D in macrophages
Source: Commun Chem. 2023 Nov 2;6:237. doi: 10.1038/s42004-023-01035-9 (PMC10622513; doi:10.1038/s42004-023-01035-9)
Supplement: Supplementary file 3 — Description of Additional Supplementary Files [file 42004_2023_1035_MOESM3_ESM.pdf]

Description of Additional Supplementary Files

File name: Supplementary Data 1

Description: Excel raw data for fig 3
