# Supplementary material for: A fluorogenic, peptide-based probe for the detection of Cathepsin D in macrophages
Source: Commun Chem. 2023 Nov 2;6:237. doi: 10.1038/s42004-023-01035-9 (PMC10622513; doi:10.1038/s42004-023-01035-9)
Supplement: Supplementary file 5 — Reporting summary [file 42004_2023_1035_MOESM5_ESM.pdf]

nature portfolio

Corresponding author(s): Mark Bradley

Last updated by author(s): Oct 14, 2023

Reporting Summary

Nature Portfolio wishes to improve the reproducibility of the work that we publish. This form provides structure for consistency and transparency in reporting. For further information on Nature Portfolio policies, see our Editorial Policies and the Editorial Policy Checklist.

Statistics

For all statistical analyses, confirm that the following items are present in the figure legend, table legend, main text, or Methods section.

n/a	Confirmed	
☐	☒	The exact sample size (*n*) for each experimental group/condition, given as a discrete number and unit of measurement
☐	☒	A statement on whether measurements were taken from distinct samples or whether the same sample was measured repeatedly
☐	☒	The statistical test(s) used AND whether they are one- or two-sided<br>*Only common tests should be described solely by name; describe more complex techniques in the Methods section.*
☒	☐	A description of all covariates tested
☒	☐	A description of any assumptions or corrections, such as tests of normality and adjustment for multiple comparisons
☐	☒	A full description of the statistical parameters including central tendency (e.g. means) or other basic estimates (e.g. regression coefficient) AND variation (e.g. standard deviation) or associated estimates of uncertainty (e.g. confidence intervals)
☐	☒	For null hypothesis testing, the test statistic (e.g. *F*, *t*, *r*) with confidence intervals, effect sizes, degrees of freedom and *P* value noted<br>*Give P values as exact values whenever suitable.*
☒	☐	For Bayesian analysis, information on the choice of priors and Markov chain Monte Carlo settings
☒	☐	For hierarchical and complex designs, identification of the appropriate level for tests and full reporting of outcomes
☒	☐	Estimates of effect sizes (e.g. Cohen's *d*, Pearson's *r*), indicating how they were calculated

Our web collection on statistics for biologists contains articles on many of the points above.

Software and code

Policy information about availability of computer code

Data collection	Plate Reader - Biotek Synergy HT multi-mode reader (λex 485/20, λem 520/20) using a 96-well plate (Life Technologies)<br>Fluorimeter - Shimatzu RF-6000<br>LC-MS - Agilent 1100<br>NMR - Bruker NMR AV500<br>Microscopy Images - Leica SP5 confocal microscope with a 63× objective<br>ESI–MS - Agilent Technologies LC/MSD Series 1100 quadrupole mass spectrometer (QMS) in ESI mode.<br>HRMS Bruker SolariX Fourier transform ion cyclotron resonance mass spectrometer (FT-MS).<br>MS spectrum collected on Bruker Ultraflextreme MALDI TOF/TOF data in of sinapic acid (10 mg/mL) in H2O/CH3CN/TFA (50/50/0.1).
Data analysis	Data plotiting and statistical analysis - Excel and OriginLab 2022 software<br>BioTeK Gen5 Software for plate reader data collection<br>Fluorimeter data collection - LabSolutions Shimatzu<br>Bruker Flexanalysis 3.4 software for MS spectrum analysis<br>MNova MestreLab for NMR spectrum analysis

For manuscripts utilizing custom algorithms or software that are central to the research but not yet described in published literature, software must be made available to editors and reviewers. We strongly encourage code deposition in a community repository (e.g. GitHub). See the Nature Portfolio guidelines for submitting code & software for further information.

Data

Policy information about availability of data

All manuscripts must include a data availability statement. This statement should provide the following information, where applicable:

- Accession codes, unique identifiers, or web links for publicly available datasets
- A description of any restrictions on data availability
- For clinical datasets or third party data, please ensure that the statement adheres to our policy

" Data supporting the paper is provided in the supplementary information section. The raw data for Fig. 3 has been provided as Supplementary Data 1."

Research involving human participants, their data, or biological material

Policy information about studies with human participants or human data. See also policy information about sex, gender (identity/presentation), and sexual orientation and race, ethnicity and racism.

Reporting on sex and gender	For primary cells derived from healthy human donor blood, cells used included donors of approximately equal numbers of both sexes.
Reporting on race, ethnicity, or other socially relevant groupings	For primary cells derived from healthy human donor blood, cells were obtained from of a mix of ethnicity but predominantly Caucasian donors.
Population characteristics	See above
Recruitment	Participants for blood donation provided written informed consent to enrol in an ethically approved project for use of cells derived from human blood in studies of the immune system. Ethical approval details provided below.
Ethics oversight	Ethical approval for use of healthy donor blood is provided by EMREC Reference number 21-EMREC-041.

Note that full information on the approval of the study protocol must also be provided in the manuscript.

Field-specific reporting

Please select the one below that is the best fit for your research. If you are not sure, read the appropriate sections before making your selection.

☒ Life sciences ☐ Behavioural & social sciences ☐ Ecological, evolutionary & environmental sciences

For a reference copy of the document with all sections, see nature.com/documents/nr-reporting-summary-flat.pdf

Life sciences study design

All studies must disclose on these points even when the disclosure is negative.

Sample size	Experiments using monocyte derived macrophages obtained from healthy donor peripheral blood were performed on a minimum of 3 separate donors, to ensure reproducibility of staining. Sample size calculation was not performed for this study, as the purpose was to obtain representative examples of probe staining in this cell type.
Data exclusions	No data were excluded
Replication	Experiments were performed on cells obtained from a minimum of 3 separate donors to obtain representative examples, which successfullly showed the reproducibility of the probe staining.
Randomization	Randomization was not required - cells from each donor were treated with all conditions shown (e.g. mock-infection, Spn infection etc.) to demonstrate reproducibility between samples.
Blinding	Blinding was not relevant to the study.

Reporting for specific materials, systems and methods

We require information from authors about some types of materials, experimental systems and methods used in many studies. Here, indicate whether each material, system or method listed is relevant to your study. If you are not sure if a list item applies to your research, read the appropriate section before selecting a response.

Materials & experimental systems

n/a	Involved in the study
☒	☐ Antibodies
☒	☐ Eukaryotic cell lines
☒	☐ Palaeontology and archaeology
☒	☐ Animals and other organisms
☒	☐ Clinical data
☒	☐ Dual use research of concern
☒	☐ Plants

Methods

n/a	Involved in the study
☒	☐ ChIP-seq
☒	☐ Flow cytometry
☒	☐ MRI-based neuroimaging

Plants

Seed stocks	n/a
Novel plant genotypes	n/a
Authentication	n/a
